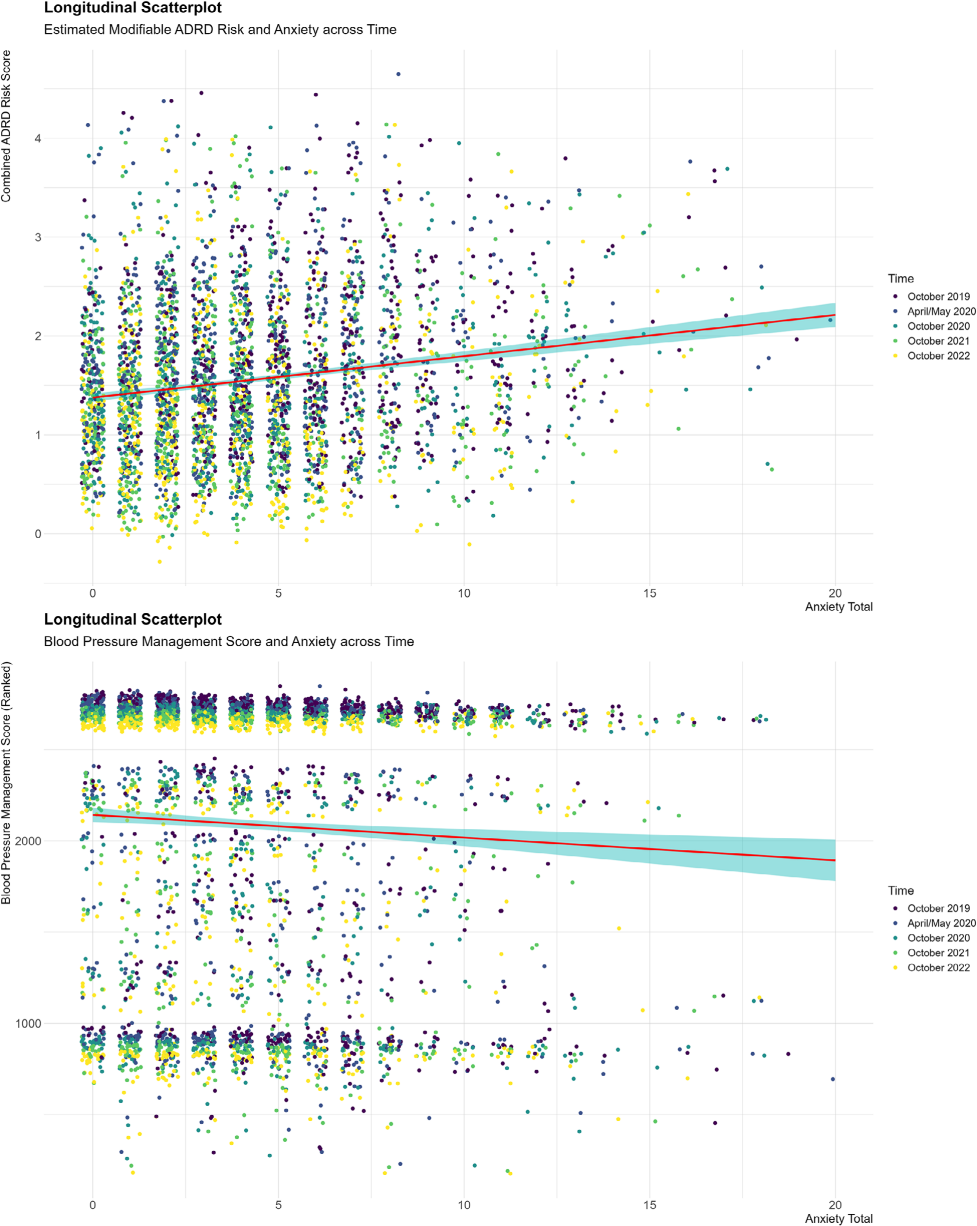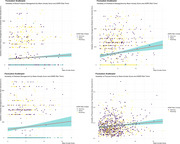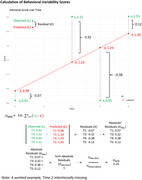# Anxiety and Anxiety‐Related Inconsistency Impacts the Modifiable Risk and Risk Behaviors for Alzheimer's Disease and Related Dementias

**DOI:** 10.1002/alz70860_107682

**Published:** 2025-12-23

**Authors:** James J.R. Brady, Larissa Bartlett, James C. Vickers, Duncan Sinclair

**Affiliations:** ^1^ Wicking Dementia Research and Education Centre, University of Tasmania, Hobart, TAS, Australia; ^2^ Menzies Institute for Medical Research, Hobart, TAS, Australia

## Abstract

**Background:**

Little is known about the impact of anxiety on the consistency of modifiable Alzheimer's disease and related dementia (ADRD) risk and risk‐related behaviors in non‐clinical older adult populations. Anxious behaviors are prone to fluctuate and may undermine efforts to lower modifiable ADRD risk. Health‐related behavior may be anxiously contradictory, either singly within one domain, or concurrently across many. Guided by well‐established stress and coping‐related frameworks, we address knowledge gaps by exploring the association between anxiety and the modifiable ADRD risk and risk‐associated behaviors of community‐dwelling older adults.

**Method:**

We analyzed data from 838 adults aged over 50 engaged in the Island Study Linking Ageing and Neurodegenerative Disease (ISLAND) in Tasmania, Australia. Initially, longitudinal ADRD risk behaviors, and a weighted ADRD risk composite score, were linearly regressed in a mixed effects model on levels of anxiety and sociodemographic covariates. Next, individualized estimates of behavioral variance across five timepoints, between 2019‐2022, were generated for nine modifiable ADRD risks. Variance scores were generated by drawing within‐participant trendlines for each ADRD risk behavior, then calculating mean absolute error scores ‐ the averaged summed distance between actual vs expected ADRD risk behaviors – representing time‐averaged strengths of participants’ behavioral fluctuations. Fluctuation scores for modifiable ADRD risks were regressed, unadjusted, on time‐averaged anxiety levels. Standardized coefficients (β) are reported.

**Result:**

Longitudinally, anxiety was significantly and negatively associated with attention to the management of blood pressure (β=‐0.04, *p* = .008), and positively associated with ADRD risk (β=0.08, *p* = .002), exceeding false discovery rate corrections (FDRs). Mean anxiety significantly and positively predicted fluctuations in ADRD risk‐related behaviors, including levels of physical activity (β=0.12, *p* < .001), and attention to the management of blood pressure (β=0.05, *p* = .001), diabetes (β=0.09, *p* < .001), and cholesterol (β=0.14, *p* < .001), which surpassed FDRs.

**Conclusion:**

This study evidenced a detrimental linear association between anxiety and blood pressure‐associated ADRD risk behavior, and overall ADRD risk over time. It revealed an association between anxiety and behavioral inconsistency across four cardiometabolic‐related ADRD risk domains. Our findings encourage nuanced investigation of anxiety's influence on modifiable ADRD risk, beyond traditional linear methods. Significant associations between anxiety and the consistency of health‐related behavior have design implications for modifiable ADRD risk‐reducing interventions within community‐dwelling older adult populations.